# Supplementary material for: Regeneration of Escherichia coli Giant Protoplasts to Their Original Form
Source: Life (Basel). 2019 Mar 1;9(1):24. doi: 10.3390/life9010024 (PMC6463199; doi:10.3390/life9010024)
Supplement: Supplementary file 1 [file life-09-00024-s001.zip › Tabata et al/Tabata etal Supplementary Figure_2.docx]

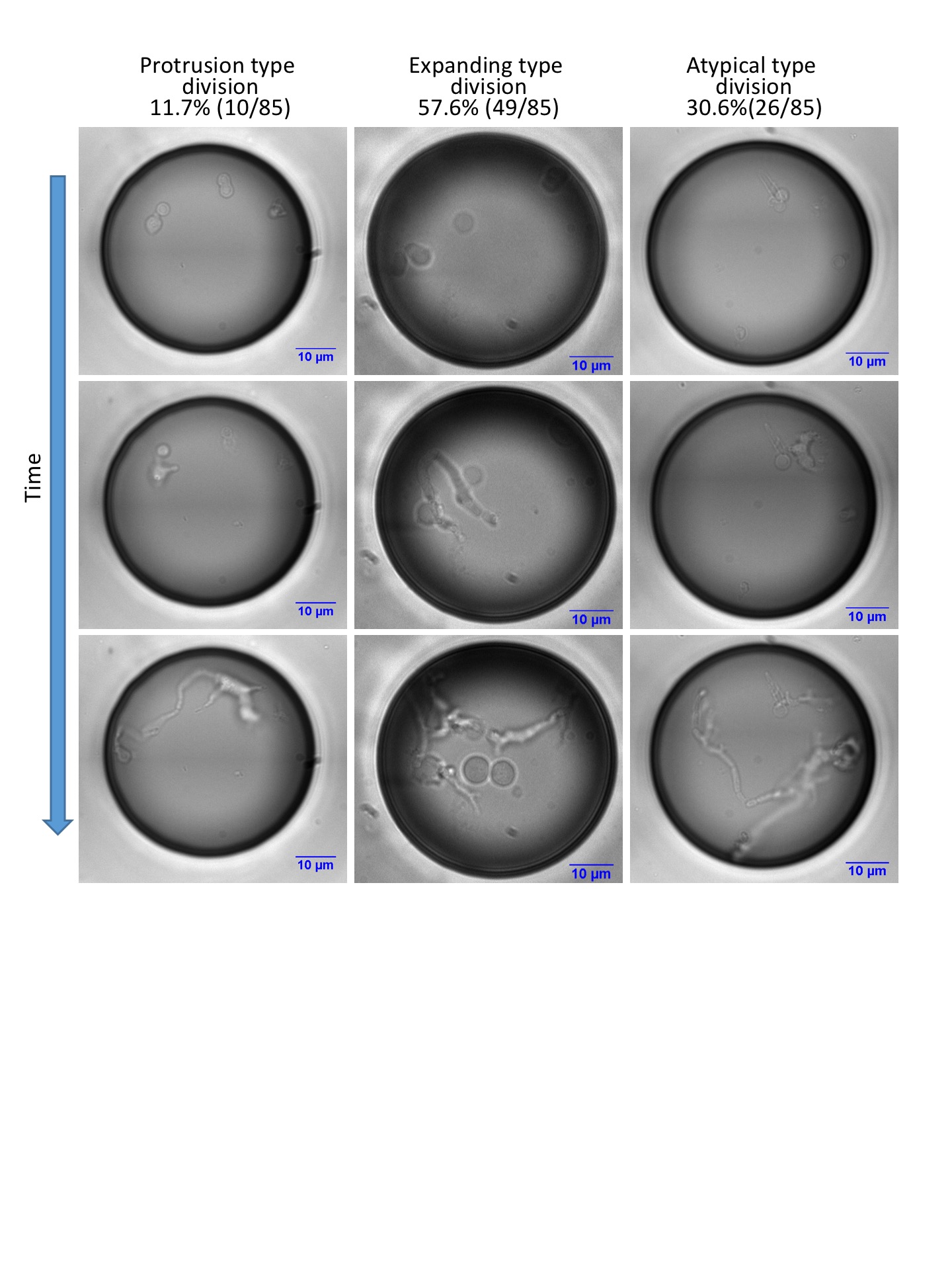


Figure S1: GP transformation and division through various forms

We classified GP that transform and divide into three categories. In protrusion type, division forms at the poles of a spherical GP, then extends and splits from it. In expanding type division, spherical GP elongate and become bacilliform before dividing. In atypical type, the division cannot be described by the other two types. The photo shows passage of time from top to bottom.


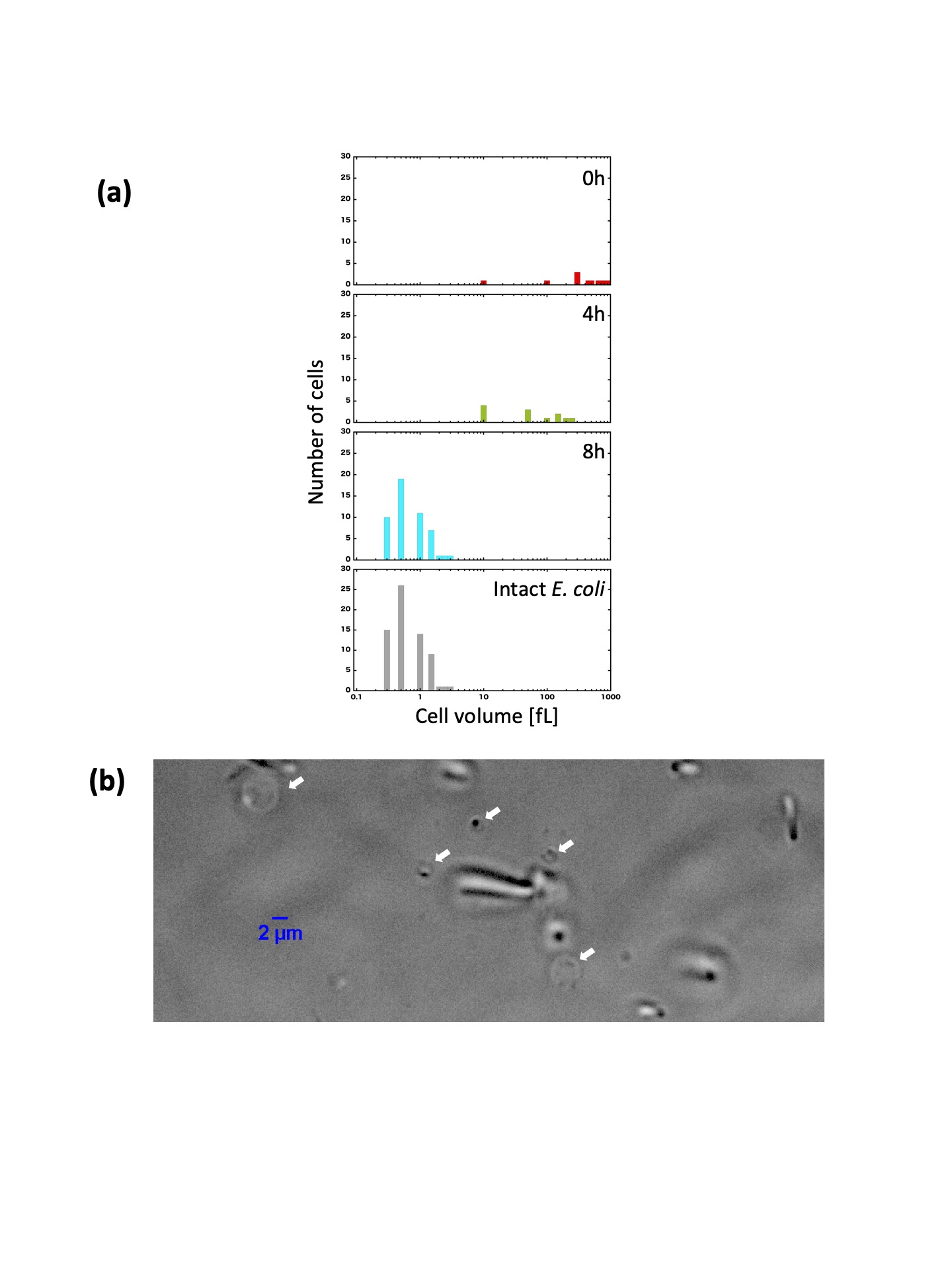


Figure S2: Relationship regenerating GP volume and WT *E. coli* volume

Histogram of temporal changes in regenerating GP volume and WT *E. coli* volume (bottom). The horizontal axis plots cell volume in logarithm. The volume was obtained at the start of the observation (0 h), 4 hours, and 8 hours, and then plotted.


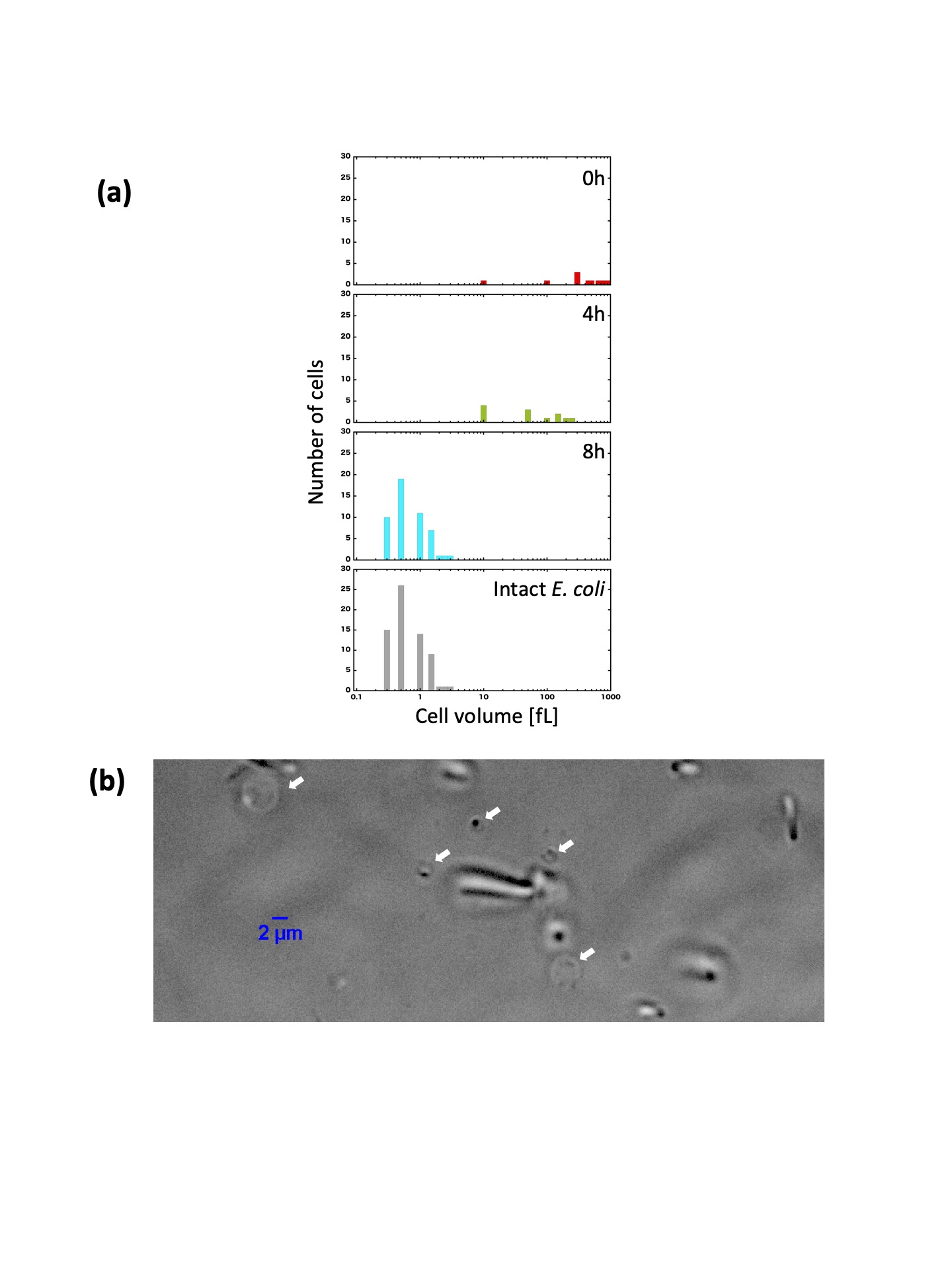


Figure S3: Microscopic image of a fried-egg like colony was suspended in the medium.

Spherical bacteria mixed with bacilli were observed (arrows).

Video S1: Localization of FtsZ-YFP in GP disruption

Bright field images and fluorescence images are merged. Yellow is fluorescence of FtsZ-YFP.
